# Hurt Feelings and Blocked Complexity in American Politics: Interpersonal Wounds Under Political Polarization and Social Distance

**DOI:** 10.3390/bs15081103

**Published:** 2025-08-14

**Authors:** Chris Kam

**Affiliations:** Department of Psychology, Adams State University, Alamosa, CO 81101, USA; ckam@adams.edu

**Keywords:** affective polarization, social distance, integrative complexity, political psychology, interpersonal relationships

## Abstract

This study assessed connections between five negative interpersonal feelings with political polarization in America. A total of 203 participants, Democrats and Republicans, were studied to see if their level of feeling hurt, dismissed, misunderstood, offended, and looked down upon was connected with their level of affective polarization, social distance, and integrative complexity. Positive correlations were found amongst all five negative interpersonal feelings and the level of affective polarization and social distance for both party members. Feeling hurt, offended, and looked down upon were negatively correlated with integrative complexity in political issues. Feeling dismissed was a predictor of lower integrative complexity. Implications for future research and real-world application are discussed in the conclusion.

## 1. Introduction

Polarization in American politics has nearly doubled from 1978 to 2020 (Tyler & Iyengar, 2024). When this happens, Democrats and Republicans can ascribe love and benevolence to their side and hatred and evil to the other (Waytz et al., 2014). In one study, 45% of U.S. college students reported reluctance to discuss politics, and over 79% shared how they were reluctant to discuss at least one controversial topic (Jones & Arnold, 2024). While there is progress in understanding political polarization from cognitive and social psychology (Balinhas, 2023), other dimensions pertaining to unconscious dimensions of relationships have been neglected (Kam & Bellehumeur, 2024). Addressing these other dimensions is crucial to ameliorate issues beyond surface level interventions. According to counseling psychology, addressing underlying hurts and wounded emotions can bring deeper healing to restore interpersonal wellness (Kam, 2023; Kam & Bellehumeur, 2021; Kam & Vriend Fluit, 2023) since healing underlying hurt and trauma can lead to interpersonal improvements (Abbass et al., 2012; Davanloo, 1987; Johansson et al., 2014; Kam, 2024; Town et al., 2013). These principles of counseling psychology apply to addressing political tensions in our day since exploring the role of negative interpersonal emotions remains largely unexplored in the literature. This study meets this gap by exploring how feelings of being hurt, misunderstood, dismissed, offended, and looked down upon relate to affective polarization (AP), social distance (SD) and integrative complexity (IC), the latter of which has shown relevance to both AP and SD.

## 2. Literature Review

Political polarization is defined as “growing ideological and policy-based differences between political groups and the affective distance between them” (Balinhas, 2023, p. 2). Within this umbrella term, there is affective polarization (AP), which involves the interpersonal and emotional elements of it. AP involves individuals perceiving others of opposing political positions with increasing hostility and emotional distance as they are viewed as untrustworthy, unintelligent, and even immoral (Iyengar et al., 2012). This happens as partisan individuals increasingly derive their political identity from group membership leading to emotional hostility, perceiving outgroup members as abhorrent, and even dehumanizing them (Finkel et al., 2020).

Social distance (SD) is defined as “the degree of sympathetic understanding that functions between person and person, between person and group, and between groups” (Bogardus, 1959, p. 7). This degree of sympathetic understanding involves emotional and psychological closeness (or the lack of it) with others. This construct is widely used in many countries for measuring prejudice towards a variety of groups (Wark & Galliher, 2007). SD has been associated with AP in the study of political polarization (Kekkonen et al., 2022).

Integrative complexity (IC) is the degree to which someone engages in differentiation of separate aspects of an issue and integrates them together. (Baker-Brown et al., 1992). Differentiation involves perceiving different dimensions of an issue. Integration involves building conceptual and interactive connections between different dimensions or perspectives of the issue. IC has shown to be highly relevant to AP. For example, the level of IC in political communications predicts how likely war will occur, where lower IC predicts that war is impending (Cervone & Pervin, 2019; Suedfeld & Tetlock, 1991). Peaceful negotiations in politics involve increased IC (Suedfeld, 2010). There has been a noted trend in recent years for U.S. presidents to have less IC in their rhetoric (Conway & Zubrod, 2022), which coincides with increased polarization in the same time frame (Tyler & Iyengar, 2024).

Higher IC is associated with better interpersonal skills, communication, effectiveness (Burleson & Caplan, 1998), increased empathy (Heck & Davis, 1973), increased tolerance for ambiguity, a lowering of psychological defenses, and better mental health and well-being (Alkoby et al., 2019; Kashdan & Rottenberg, 2010). All these factors associated with IC can lead to lower AP and SD in interpersonal interaction, whereas their absence can increase AP and SD.

In the domain of counseling psychology, five qualities that indicate a lack of interpersonal health and wellness are feeling hurt, misunderstood, dismissed, offended, and looked down upon. All of these feelings are relational in nature and involve two or more people in interpersonal interaction. As a result, they can be conceptualized as being involved in affective polarization and social distance since AP and SD are also interpersonal in nature. Since these five qualities have been known to affect the quality of interpersonal relationships, there is a need to study if there is a correlation between them and AP as well as SD. With regard to these five qualities themselves, past research has shown how each of them affect the quality of bond between two or more people interacting with each other.

Individuals feel distant from others after feeling hurt by them (Vangelisti, 2001), harbor hostility and anxiety as a result (Leary et al., 1998), and are less satisfied in hurt relationships overtime (Vangelisti & Redlick, 2017). Feeling misunderstood can lead to one’s personhood feeling rejected by others (Green, 2023), feeling discordant with them (Condon, 2010), and feeling relentless frustration (Gaillard et al., 2009). When individuals feel dismissed by someone, it leads to invalidating their embodied realities which can lead to prolonged negative emotional and psychological states (Ruzicka, 2013) and can also lead to disengagement (Kwint, 2024). When feeling offended, people can feel inferior with a loss of self-esteem (Poggi & D’Errico, 2018) and feel that it is harder to maintain empathy and a nonjudgmental stance towards the other (Ahn et al., 2021). Furthermore, feeling offended can trigger emotions of anger, bitterness, and rancor towards the perceived offender, breaking the relationship (Poggi & D’Errico, 2018). With regard to condescension, prestige-motivated grandstanding is linked to ideological extremism in political polarization (Grubbs et al., 2020) and contempt, which is associated with potential anger, disgust, and physical assault in relationships (Gervais & Fessler, 2017; Sommer et al., 2016). Feelings of superiority in comparison to others can lead to relational distance (van Osch et al., 2017), which is related to the constructs of AP and SD.

With these findings taken into consideration, there is a need for research examining the relationship between these forms of relational distress and pain with AP, SD and IC. This addresses a gap in the literature and may shed more insight on how to counter AP and SD on a collectively deeper level in national political discourse.

This study seeks to find answers to the question of whether these five factors of feeling hurt, misunderstood, dismissed, looked down upon, or offended by the other political party are connected to AP, SD, and IC in American politics with respect to the two major parties.

The hypotheses of this study are as follows:

**H1.**  *There will be a positive correlation between feeling hurt, dismissed, misunderstood, offended, and looked down upon by the other political party and AP level.*

**H2.**  *There will be a positive correlation between feeling hurt, dismissed, misunderstood, offended, and looked down upon by the other political party and SD level.*

**H3.**  *There will be a negative correlation between feeling of being hurt, dismissed, misunderstood, offended, and looked down upon by the other political party and IC level.*

**H4.**  *There will be a negative correlation between IC level and AP level.*

**H5.**  *There will be a negative correlation between IC level and SD level.*

## 3. Methods

### 3.1. Study Size

A priori power analysis was calculated using G*Power 3.1.9.7 (Faul et al., 2009) to determine what sample size was required for a medium correlation between variables. A size of 84 was required for a two-tailed test, with α = 0.05 and power = 0.80. Taking into account multiple predictors and a Bonferroni-corrected α = 0.01 (for five tests), the required sample size increased to 125. Targeting 200 participants accounted for 10–15% attrition while ensuring enough power for multiple correlations.

### 3.2. Setting

The research design was approved by the IRB of Adams State University (IRB Record# 2025-5-02R). Participants were recruited through the research website Prolific on 22 May 2025. They completed the survey questions and were compensated USD 2.00 for approximately 10–15 min of their time. Participants had to be 18 years old or above and have an American citizenship to participate. Since this was a study specifically on political polarization, in line with many studies on the subject, it specifically targeted self-identifying Republican and Democrats; those who did not self-identify as either were screened out. Participants accessed the Qualtrics survey via Prolific and read the terms and conditions of the study. The length of the study and description of its components were outlined to participants. If they agreed to take the study, they were told that they could voluntarily drop out at any time. Consenting participants were told that their input would be stored in a secure place used for research purposes only and that their input may be anonymously used for solely for research purposes.

### 3.3. Participants

A total of *n* = 203 participants were recruited. There were 100 self-identifying Democrats and 103 self-identifying Republicans. In this sample, 41.6% were male, and 57.4% were female; 66% were White, 24.1% were African American, 4.9% were Hispanic, 3.9% were Asian, and 1% were Native American or Alaska Native. Two participants were screened out since they indicated no political affiliation. One set of data was discarded since the participant copied and pasted a question into the text box for answering. Since the participants of the study were recruited through an online research platform, Prolific, it was not a purely randomized sample of the U.S. population. On the one hand, a quota system was used so that there was approximately a 1:1 ratio for Democrats and Republicans recruited that avoided a pure convenience sampling method since Prolific consists of individuals of various backgrounds from over 30 countries choosing to sign up with the platform. On the other hand, this was a non-probability sample that does not have the full generalizability of a pure random sample since not everyone is signed up with the research website.

### 3.4. Quantitative Variables and Measurement

*Measuring Affective Polarization.* The American National Election Studies (ANES) Feeling Thermometer has been used in a reliable manner to rate Democrats and Republicans in terms of their emotional warmth (or lack thereof) towards politically involved individuals since the 1970s (Tyler & Iyengar, 2024). Over the years, participants have been asked to indicate how warm (100) or cold (0) they feel towards the other political party. The number they give the other party compared to the number they give towards their own party is computed for an AP score.

*Measuring Social Distance.* The Bogardus Social Distance scale (Bogardus, 1932) has been used for many decades to measure the level of emotional distance someone feels towards others who belong to an outgroup. It has become a standard valid and reliable way to quantify the emotional distance one feels towards outgroup members (Parrillo & Donoghue, 2013; Wark & Galliher, 2007). This study used the following prompt in this form: “*In this section, we are interested in your feelings about social interactions with people who hold political views opposite to your own—specifically, ‘those on the other side of the political aisle.’ Answer based on your general feelings, imagining a typical person from this group. There are no right or wrong answers—just respond honestly. What is the closest relationship you would be willing to have with a member of the other political party? Select one option. Choosing an option means you also accept all options below it”.* Participants chose one option out of the following in order: (1) Marriage or have as a close relative by marriage; (2) Have as a close personal friend; (3) Have as a neighbor on my street; (4) Have as a co-worker in my occupation; (5) Have as a citizen in my country; (6) Have as a visitor to my country (non-citizen); (7) Excluded from my country. The participants’ choice with its corresponding number measures the level of social distance the participant feels towards the outgroup member.

*Measuring level of feeling hurt, dismissed, misunderstood, offended, and looked down upon.* The visual analogue scale (VAS) has been used to measure many types of subjective phenomena in the psychological literature for a wide range of applications in clinical and research contexts (Lesage et al., 2012; McCormack et al., 1988; Wewers & Lowe, 1990; Yeung & Wong, 2019). It has been used since the 1920s to measure somewhat intangible qualities such as pain, anxiety, and quality of life (Freyd, 1923). Since the 1960s, it has been used to measure mood disorders (Heller et al., 2016). In some contexts, it can overcome some limitations of Likert-type and ranking scales (Sung & Wu, 2018). The VAS in this study measured each participant’s level of feeling hurt, dismissed, misunderstood, offended, and looked down upon on a scale of 0 (not at all) to 100 (extremely). They were presented with the following prompt: “On a scale of 0 (not at all) to 100 (extremely), how much do you feel [blank] by the other political side?”

*Measuring IC.* Participants were instructed to freely write their thoughts with the following prompt: “*Please write 2–3 paragraphs about your thoughts on a political issue currently in the news. Feel free to share whatever comes to mind, such as the issue’s significance, different perspectives on it, or potential ways to address it*”. IC level was assessed with the Automated Integrative Complexity (AutoIC) scoring system (Conway et al., 2014; Conway et al., 2020; Houck et al., 2014). This automated scoring system assesses both differentiation (acknowledging multiple perspectives) and integration (connecting those different perspectives together) levels as human coders would in the same hierarchical manner to produce a numerical representation of how complex a paragraph’s IC is. To create this scoring program, experts trained in IC scoring performed linguistical analysis of all words and phrases associated with integratively simple or integratively complex language involving synonym trees (involving over 3500 complexity-related words and phrases). After creating a system from this process, researchers trained it on a set of data using expert human scoring of IC as a benchmark. AutoIC has high correlations with expert human scorers and replicates human scoring caliber (Conway et al., 2020). It has been utilized to analyze IC levels of U.S. presidents (Conway & Zubrod, 2022) as well as Trump and Biden supporters (Abe, 2022). AutoIC assesses the level of IC from 1 to 7 (with 7 having the highest levels of IC).

## 4. Results

Statistical analyses were completed with *Jamovi* statistical software (Version 2.6.26; The Jamovi Project, 2025). Correlational tests were carried out with the all the measured variables in the study to test all hypotheses. In addition, linear regression calculations were performed as well as a *t*-tests to compare scores on the measured variables between Democrats and Republicans.

Table 1 has the mean scores for each measure for Democrats and Republicans.

Independent *t*-tests showed Democrats reporting significantly higher levels of feeling hurt, dismissed, misunderstood, offended, and looked down upon compared to their Republican counterparts, all with *p* < .001.

Other *t*-tests also showed that Democrats reported higher AP (M = 55.7) than Republicans (M = 47.1, *t*(df) = 2.18, *p* < .05). Democrats also expressed slightly higher social distance (M = 3.10, *t*(df) = 1.16, *p* = 0.249) compared to Republicans (M = 2.83) but this was not statistically significant. Republicans scored slightly higher on IC (M = 2.69) than Democrats (M = 2.27, *t*(df) = −2.25, *p* < .05).

In terms of the correlational analyses performed for the hypotheses, H1 and H2 were supported, while H3 was only partially supported. H4 and H5 were not supported by the data.

Table 2 summarizes the correlational data between key measured variables of the study.

Hypothesis 1 was supported by the data:

**H1.**  *There will be a positive correlation between feeling hurt, dismissed, misunderstood, offended, and looked down upon by the other political party and AP level.*

AP was positively correlated with all five interpersonal emotions—hurt (*r* = .435), misunderstood (*r* = .407), dismissed (*r* = .499), offended (*r* = .475), and looked down upon (*r* = .510), all at *p* < .001.

**H2.**  *There will be a positive correlation between feeling hurt, dismissed, misunderstood, offended, and looked down upon by the other political party and social distance (SD) level.*

SD had a positive correlation with all five interpersonal emotions (range *r* = .234 to .296, *p* < .001), albeit to a lower degree than with AP.

Hypothesis 3 was partially supported by the data:

**H3.**  *There will be a negative correlation between feeling hurt, dismissed, misunderstood, offended, and looked down upon by the other political party and IC level.*

There was a negative correlation between IC and feeling hurt (*r* = −.159, *p* = .024), offended (*r* = −.144, *p* = .041), and looked down upon (*r* = −.204, *p* = .004) with significance. However, even though there were negative correlations between IC and feeling dismissed (*r* = −0.120, *p* = 0.089) and feeling misunderstood (*r* = −0.093, *p* = 0.187), these relationships were not at significant levels.

**H4.**  *There will be a negative correlation between IC level and AP level.*

This hypothesis was not supported by the data. The negative correlation between IC and AP was minimal and insignificant (*r* = −0.066, *p* = 0.349).

**H5.**  *There will be a negative correlation between IC level and SD level.*

This hypothesis was also not supported by the data. The negative correlation between IC and SD was also minimal and insignificant (*r* = −0.002, *p* = 0.973).

Additional correlational analyses were carried out with age and measured variables of the study. Age was correlated with AP (*r* = 0.23, *p* = 0.001), SD (*r* = 0.207, *p* = 0.003), feeling hurt (*r* = 0.155, *p* = 0.027), feeling misunderstood (*r* = 0.148, *p* = .035), feeling dismissed (*r* = 0.182, *p* = .010), and feeling offended (*r* = 0.203, *p* = .004).

Multiple linear regression calculations were also performed to predict IC after taking into account demographic factors such as age, gender, ethnicity, education, political identification, as well as AP, SD, level of political interest, and the five measured interpersonal measures of the study. Feeling looked down upon was found to be a significant predictor of IC. The higher a participant’s level of feeling looked down upon, the lower their predicted level of IC (Estimate = −0.0109, SE = 0.0050, t = −2.16, p = 0.032). Other linear regression calculations using other predictor variables in the study were not significant.

## 5. Discussion

There are many findings worth reflecting upon in this study. There were significant correlations (i.e., *p* < .001) for all of the five interpersonal feelings explicitly measured in this study (i.e., feeling hurt, dismissed, misunderstood, offended, and looked down upon) with both AP and SD. This is an important finding to inform the larger conversation on countering political polarization in America. These findings suggest that there is a need to address these interpersonal emotions in the political process in order to address the partisan divides in the country. Previous interventions that focused on more cognitive and exposure oriented means are still important but may have limits if they do not address underlying interpersonal hurts in the interaction process from both major political parties. It is possible that utilizing counseling-like interventions such as compassionate validating, outward signs of respect, and helping others feel seen and honored may possibly provide some of the conditions for bridging the emotional elements of the political divides and behavioral distance in the country between people who disagree. Future studies can test whether these types of interventions are effective in countering AP and SD in the political context.

The data showing that feeling hurt, offended, and looked down upon is significantly associated with IC in a negative direction is also important to consider in healing the country’s political tensions. Since IC has shown to be an important factor in addressing AP (Kam & Bellehumeur, 2024), and AP has been shown to be connected with SD (Kekkonen et al., 2022), these findings are insightful to consider when aiming to increase IC to counter polarization. It could be that feeling hurt, offended, and looked down upon blocks individuals from engaging in controversial political topics with more IC. This could be because emotional pain can block curiosity and the capacity to hold sophisticated dimensions of an issue in tension with ambiguity. When this ability is blocked, it can lead to the processing of complex issues in a very simplistic manner. In contrast, it could also be the case that having higher IC can protect one from feeling hurt, offended, and looked down upon since higher IC can prevent all-or-nothing attitudes that exacerbate feeling hurt, offended, or looked down upon. With regard to feeling dismissed and misunderstood, although these were also negatively correlated with IC, it was not at significant levels. More research has to be done to see if certain versions of feeling dismissed or misunderstood coexist with a lack of IC or not.

Interestingly, there was no significant negative relationship between IC and AP or SD (what Hypotheses 4 and 5 tested). It is not clear why this was not found even though previous research has supported how IC and AP are negatively associated in a significant way (Conway et al., 2001; Conway et al., 2016; Conway et al., 2018; May & Zekilow, 1997; Suedfeld, 2010; Tetlock et al., 1994; Wallace & Suedfeld, 1988). More study should be conducted to examine which conditions allow IC and AP to be negatively correlated with significance and which do not. Similar things can be said about the potential negative relationship between IC and SD, since AP and SD have been shown to be empirically related. It could be that some individuals can separate the complexity of issues with their personal hurts, while others have trouble doing so. It could also be that one’s level of IC may be compartmentalized towards certain subdomains of political issues, where some issues are processed with more complexity than others. This study had a general prompt that invited participants to write about whatever political issues came to mind without prompting them to write about issues where they had more (or less) IC. Future studies can examine these empirical nuances.

Another intriguing finding was how age was positively correlated with feeling hurt, misunderstood, dismissed, and offended at significant levels. As there was no significant difference in age between Democrat and Republican participants in the study (*F*(1, 201) = 1.17, *p* = .24), this finding seems to be a pattern across party lines. There are several possible implications of this. One is that as one ages, one has more exposure to hurtful situations in politics. Another is that young people may be less sensitive to interpersonal slights from others in political discourse, while older individuals are more sensitive. It could also be the case that when expectations of a baseline level of respect and civility in older individuals interacting are violated, it impacts them more than when younger individuals violate these expectations towards each other. Still, it could be the case that older individuals have accumulated more associations between certain stimuli and interpersonal slights (e.g., slogans from the other side with demeaning attitudes towards them) that trigger greater emotional impact than for younger individuals. This correlation between age and feeling hurt, misunderstood, dismissed, and offended is in contrast to the variable of formal education, which was not significantly related to the measured variables of the study. There are some possibilities for this. One is that formal education may not be a relevant factor affecting how hurt one is in relation to the other political party. Another could be that formal education does not help heal hurt feelings in the political context. More research can be done to explore this lack of connection here, as education may have some potential to heal partisan divides if approached with a certain mindset.

Feeling looked down upon being a significant predictor of IC (Estimate = –0.0109, SE = 0.0050, t = –2.16, p = 0.032) is another finding that merits further study. The observation that the more someone feels looked down upon, the more likely they are to have lower IC in political issues is something for educators and policy legislators to take into account going forward. Since feeling looked down upon can negatively predict IC, the opposite condition can be tested, where feeling respected or even honored can increase IC in political discourse. Future studies can empirically test this. With this possibility in mind, universities may be able to see if they can increase the level of IC in their classrooms by helping students feel more respected and even honored as they disagree on sensitive and politically controversial topics on campus.

It should be noted that since this study was conducted after the 2024 U.S. presidential election, where Republicans won and the Democrats lost, the timing of this study (May 2025) could affect Democrats’ experience and/or self-report of all the measured variables (i.e., AP, SD, IC, and all the five relational emotions measured). Future studies conducted at different times, such as the mid-term elections or time periods with distance from any major elections, can potentially complement the findings in this study.

There are also other limitations to this study. Caution must be applied in interpreting the results, as this study was merely correlational and did not study causation. This study alone cannot determine whether feeling hurt, dismissed, misunderstood, offended, or looked down upon causes AP, SD, and in some cases lower IC or whether it is the other way around. These assertions must merit experimental support to hold water. Future directions for study should include ethically sound experimental procedures to see if these five interpersonal feelings induce higher AP, SD, or lower IC or the other way around.

In terms of demographics, no self-identified Independents were studied here. Doing so in future studies could lead to more nuance in understanding how these five interpersonal emotions and AP, SD, and IC all interact. Future studies can be designed to research whether AP, SD, or IC are correlated with the five interpersonal emotions in this study for self-identifying Independents, “centrists”, and also so-called “moderates”. Doing so would lead to a more complex characterization of a significant portion of the American electorate with respect to these variables. Also, in future studies, non-Americans in other countries can be studied to see if correlations similar to or different from those found in this study are also found elsewhere since the particular style of American politics is not ubiquitous around the world.

Additional limitations of this study include the information being gathered only online. While this allowed for a potentially broader sample, it did not allow for the monitoring of the distraction levels of participants on their devices while competing the questions. Conducting in-person studies can rectify this potential issue in future studies. Additionally, all the measures were carried out through self-report, which can open the door to personal biases in participants’ own self-concepts and limitations in self-awareness. Future studies can rectify this by triangulating the data through third-party assessments (e.g., friends or family members who know the participants well) to provide a more holistic assessment of how participants experience these five interpersonal emotions as well as AP, SD, and IC in politics.

Another direction for future research can be qualitative means of studying relationships between the measured variables in this study. Semi-structured interviews and focus groups can be conducted to study the interaction of these variables in a more phenomenological manner. Addressing the subjective experience of how these phenomena interact can inform future models to provide heightened explanatory power in a testable way.

In conclusion, this study provides the groundwork for future studies examining how feeling hurt, dismissed, misunderstood, offended, and looked down upon can interact with AP, SD, and IC. There are not only implications for future study design but for real-world interventions as well. Educators, social leaders, policy makers, and even politicians themselves can glean from these initial findings for direction in helping to heal partisan divides in the country. Instead of only directly addressing AP, SD, and IC with “Band-Aid” surface solutions, addressing these five interpersonal emotions on a deeper level may indirectly but powerfully heal underlying issues and deeper wounds blocking the healthy recovery of the body of American politics.

## Figures and Tables

**Table 1:** Group Differences in Emotional Experience and Key Variables by Political Identification.

Variable	Democrats (*n* = 100)	Republicans (*n* = 103)	t	df	*p*
Hurt	61.3	36.8	5.72	201	<.001
Misunderstood	69.6	55.0	3.82	201	<.001
Dismissed	74.1	55.3	4.63	198	<.001
Offended	61.7	45.1	3.59	200	<.001
Looked down upon	66.9	50.5	3.62	201	<.001
Affective polarization (AP)	55.7	47.1	2.18	201	<.05
Social distance (SD)	3.10	2.83	ns	—	—
Integrative complexity (IC)	2.27	2.69	−2.25	201	<.05

Note. All scores except for SD were measured from 0 to 100 (lowest to highest respectively). All *p*-values are two-tailed. ns = not significant.

**Table 2:** Correlations Among Key Variables.

Variables	AP	SD	Hurt	Misunderstood	Dismissed	Offended	Looked Down Upon	IC	Age
AP	-								
SD	.304 ***	-							
Hurt	.435 ***	.234 ***	-						
Misunderstood	.407 ***	.276 ***	.576 ***	-					
Dismissed	.499 ***	.278 ***	.612 ***	.717 ***	-				
Offended	.475 ***	.296 ***	.727 ***	.637 ***	.67 ***	-			
Looked Down Upon	.510 ***	.283 ***	.617 ***	.686 ***	.771 ***	.714 ***	-		
IC	−.066	−.002	−.159 *	−.093	−.12	−.144 *	−.204 **	-	
Age	.230 **	.207 **	0.155 *	0.148 *	0.182 *	.203 *	.122	.013	-

* *p* < .05; ** *p* < .01; *** *p* < .001.

## Data Availability

The data that support the findings of this study are available in the following database: DOI 10.17605/OSF.IO/R4536. It can be accessed with the following link: https://osf.io/g97cs/files/osfstorage, accessed on 28 May 2025.
